# Metabolic Dysfunction-Associated Steatotic Liver Disease and Alcohol-Associated Liver Disease: Liver DNA Methylation Analysis—A Systematic Review

**DOI:** 10.3390/cells13221893

**Published:** 2024-11-16

**Authors:** Daniela Stols-Gonçalves, Abraham S. Meijnikman, Luca Schiliró Tristão, Clara Lucato dos Santos, Nerissa P. Denswil, Joanne Verheij, Wanderley M. Bernardo, Max Nieuwdorp

**Affiliations:** 1 Department of Internal and Vascular Medicine, Amsterdam University Medical Centre, Meibergdreef 9 (Room A01-112), 1105 AZ Amsterdam, The Netherlands; a.s.meijnikman@amsterdamumc.nl (A.S.M.); m.nieuwdorp@amsterdamumc.nl (M.N.); 2 Department of Evidence-Based Medicine, Faculdade de Ciências Médicas de Santos—Lusiada University Center, Santos 11050-071, SP, Brazil; lucatristao@gmail.com (L.S.T.); claralucato@gmail.com (C.L.d.S.); wmbernardo@usp.br (W.M.B.); 3 Medical Library, Amsterdam University Medical Centre, University of Amsterdam, 1012 WP Amsterdam, The Netherlands; n.p.denswil@amsterdamumc.nl; 4 Department of Pathology, Amsterdam University Medical Centre, 1105 AZ Amsterdam, The Netherlands; j.verheij@amsterdamumc.nl; 5 Faculdade de Medicina d Universidade de São Paulo, São Paulo 05508-220, SP, Brazil

**Keywords:** alcohol-associated liver disease, epigenetics, insulin resistance, metabolic dysfunction-associated steatotic liver disease, peroxisome proliferator-activated receptor

## Abstract

Background: Metabolic dysfunction-associated liver disease (MASLD) and alcohol-associated liver disease (ALD) are among the leading causes of liver disease worldwide. The exact roles of epigenetic factors in both diseases remains largely unknown. In this context, liver DNA methylation remains a field that requires further exploration and understanding. Methods: We performed a systematic review of liver DNA methylation in humans with MASLD or ALD using Ovid MEDLINE, Ovid Embase, and Cochrane Library. We included human studies where liver DNA methylation was assessed in patients with MASLD and/or ALD. The Rayyan platform was used to select studies. Risk of bias was assessed with the “risk of bias in non-randomized studies of interventions” tool, ROBINS-I. We performed pathway analysis using the most important differentially methylated genes selected in each article. Results: Fifteen articles were included in this systematic review. The risk of bias was moderate to serious in all articles and bias due to confounding and patient selection was high. Sixteen common pathways, containing differentially methylated genes, including cancer pathways, were identified in both diseases. Conclusions: There are common pathways, containing differentially methylated genes, in ALD and MASLD, such as pathways in cancer and peroxisome proliferator-activated receptor (PPAR) signaling pathways. In MASLD, the insulin signaling pathway is one of the most important, and in ALD, the MAPK signaling pathway is the most important. Our study adds one more piece to the puzzle of the mechanisms involved in steatotic liver disease.

## 1. Introduction

Metabolic dysfunction-associated steatotic liver disease (MASLD) and alcohol-associated liver disease (ALD) are two significant pathological entities, each characterized by a range of similar histopathological features.

The histopathological characteristics of MASLD, according to Clinical Research Network (CRN) staging, include the following: (1) steatosis: 0: <5%; 1: 5–33%; 2: 34–66%; 3: >66%; (2) lobular inflammation: 0: none; 1: <2 foci/20× field; 2: 2–4 foci/20× field; 3: >4 foci/20× field; and (3) ballooning degeneration: 0: none; 1: few; 2: many [1]. Although MASLD and ALD show substantial overlap regarding these histomorphological features, ALD more frequently presents with bilirubinostasis, influx of neutrophils and Mallory–Denk bodies, as acknowledged in the SALVE scoring system [2]. Fibrosis is categorized in four stages in both systems, with slight differences between MASLD and ALD fibrosis patterns: in ALD, the extent of pericellular fibrosis is more pronounced, together with fibro-obliteration of hepatic veins, perivenular fibrosis and sclerosing hyaline necrosis, which is not reflected in the MAFLD staging systems.

With a global prevalence of 30% for MASLD and 5% for ALD, these diseases are at the forefront of liver-related health issues worldwide [3,4].

Addressing the distinctions between alcohol-induced and metabolic disease-induced liver injuries, a multi-society panel has advocated for the term MASLD over traditional nonalcoholic fatty liver disease (NAFLD) [5]. MASLD is defined as hepatic steatosis identified through imaging or biopsy, co-occurring with cardiometabolic risks, such as elevated BMI, insulin resistance, or dyslipidemia, in the absence of a significant alcohol history [5,6]. It can progress to metabolic dysfunction-associated steatohepatitis (MASH), marked by steatosis along with inflammatory infiltrates and ballooning degeneration, with or without the presence of Mallory–Denk bodies or pericellular fibrosis, as determined by liver biopsy. Those meeting the MASLD criteria but with notable alcohol consumption are diagnosed with metabolic alcohol-associated liver disease (MetALD). This nuanced classification underscores the intricate interplay between metabolic and alcohol-related factors in liver disease, highlighting the importance of a comprehensive approach to diagnosis and treatment to effectively address the diverse manifestations of liver injury.

Despite the substantial health and societal impact of these conditions, only one therapy for MASLD has received approval after decades of research [7]. In contrast, for ALD, especially the most severe deadly form, alcohol-associated hepatitis, there has been no approval of therapeutic interventions for over fifty years [8]. Furthermore, there is an urgent need for non-invasive biomarkers to facilitate early diagnosis, assess treatment efficacy, and determine the staging of both diseases [9].

Recent shifts in research focus have highlighted changes in the epigenetic landscape as a potential mechanism underlying the pathogenesis, maintenance, and progression of MASLD and ALD [10,11]. This includes the exploration of novel diagnostic markers. DNA methylation is the most studied epigenetic modification; it is the addition of a methyl group (CH_3_) to a cytosine base followed by a guanine base (CpG) in the DNA. It regulates gene expression and it has been previously studied in other metabolic diseases such as type 2 diabetes and obesity [12]. Despite advancements in scientific understanding, the specific role of DNA methylation in liver tissues, particularly in distinguishing between and understanding the similarities between the two main drivers of chronic liver disease worldwide, MASLD and ALD, remains unclear. Consequently, our objective was to conduct a systematic review to assess the significance of epigenetic modifications in liver tissue, specifically DNA methylation, in MASLD and ALD.

## 2. Methods

### 2.1. Search Strategy

**Figure 1:**
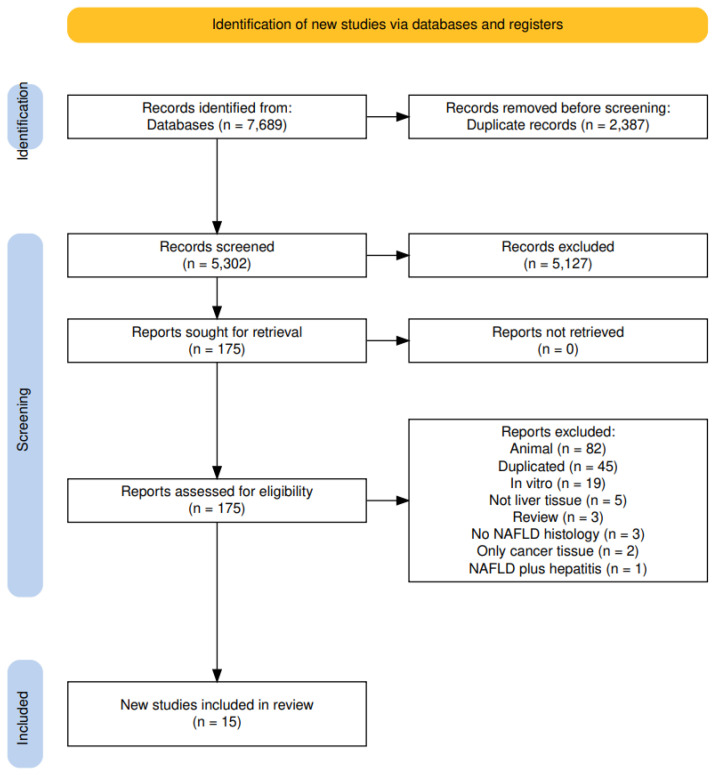
PRISMA flow diagram.

We searched MEDLINE (1946 to date) and Embase (1947 to date) using the OVID platform and Cochrane Library databases (Issue 2) from inception to 15 July A search strategy was developed for each database using (controlled) terms for the following relevant search concepts: “DNA Methylation”, “Epigenetics” and “Fatty liver disease”. The search terms were combined using the Boolean operators AND and OR. No time limit is applied. Duplicates of the search results were removed using Dedupendnote (version 1.0.1) before study selection [13]. A pre-established protocol was deposited in the International Prospective Register of Systematic Reviews (PROSPERO) [14] and followed the Preferred Reporting Items for Systematic Reviews (PRISMA-2020) guidelines [15] (register: Prospero 2024, CRD42024511974). See Figure 1.

### 2.2. Eligibility Criteria

After removing duplicates, we included manuscripts according to the following inclusion criteria: (1) human studies in all languages; (2) observational or interventional studies where specific liver DNA methylation (l-DNAm) of 5-methylcytosine (5mC) was associated with MASLD and/or ALD (both targeted l-DNAm (methylation occurring within pre-selected DNA regions) and untargeted (methylation occurring in a large portion of DNA also known as genome-wide) studies were included); (3) outcomes involving liver steatosis with or without steatohepatitis and fibrosis attributed to MASLD or ALD where other liver diseases were excluded.

### 2.3. Data Collection

Two authors independently reviewed the references identified during the search. The Rayyan platform was used to select the studies [16]. In the first selection, all titles and abstracts were read, and clearly non-eligible studies were excluded. The full texts of the remaining articles were then checked for eligibility. When there was a discrepancy between eligibility selections, a third author was consulted.

### 2.4. Data Extraction

The following data were extracted from the articles (if available): DOI, authors, year of publication, sample size, age, sex, BMI, tobacco use, alcohol intake, T2D, co-medication, other concomitant diseases, preoperative diet, DNA isolation/extraction method, DNA methylation data processing, estimation of cell proportion, and RNA seq confirmation. Genes regarded by the original authors as the most important differentially methylated genes were identified.

### 2.5. Risk of Bias Assessment

Bias analysis was performed using ROBINS-I [17], considering confounding, selection, interventions, missing data, outcomes measures, and results. The risk of bias was classified as low, moderate, or high, and the quality of evidence was estimated directly from this risk.

### 2.6. Pathway Analysis

Pathway analysis was performed using the Kyoto Encyclopedia of Genes and Genome (KEGG) using KEGG Mapper (https://www.genome.jp). We extracted the pathways in which a minimum of three differentially methylated genes were involved.

## 3. Results

### 3.1. Article Selection, Study Characteristics

#### 3.1.1. Article Selection

The search carried out until July 2023 retrieved, after duplicate exclusion, 5302 articles, of which 175 were selected for full-text evaluation. In agreement with the eligibility criteria, 15 articles were included in this systematic review [18,19,20,21,22,23,24,25,26,27,28,29,30,31,32] (Figure 1).

#### 3.1.2. Study Characteristics

Among the 15 studies included (See Table 1), age differences between groups were not statistically significant in the majority of the studies, except in one [27]. Five studies either did not report age or relied on data from larger studies [20,21,23,26,28]. Most studies found BMI to be similar between groups, with two exceptions: one noted a significant difference between the MASLD group and the “normal liver” group (*p* = 0.03) and another found that the “normal liver” group did not include obese patients (*p* = 0.0001) [29,32]. One study did not have BMI data for the “normal liver” group [27]. The criteria for a “normal control group” varied significantly. Two studies defined “normal control” as biopsies from patients undergoing surgery for benign or malignant hepatic tumors, with samples taken away from the tumor areas [21,27]. Other studies relied on a database of normal l-DNAm values from previous studies [19,25].

### 3.2. Differentially Methylated Genes in MASLD, Differentially Methylated Genes in ALD, and Differentially Methylated Genes in MASLD and ALD

#### 3.2.1. Differentially Methylated Genes in MASLD

As mentioned before, targeted l-DNAm (methylation occurring within pre-selected DNA regions) and untargeted l-DNAm (methylation occurring in a large portion of DNA, also known as genome-wide) were included.

Target l-DNAm was performed in seven MASLD papers [21,23,25,26,27,29,32]. One study showed that the promoter region in the peroxisome proliferator-activated receptor gamma, Coactivator 1alpha ***PPARGC1A,*** was hypermethylated compared to controls [32]. Another study examined the mitochondrially encoded ***MTND6***, in which hypermethylation was associated with more severe fibrosis [29].

Untargeted l-DNAm was performed in six MASLD papers [18,19,22,24,30,31]. One study compared l-DNAm in MASLD with severe fibrosis to a liver with MASLD with no histological evidence of fibrosis, resulting in seven hypomethylated CpGs associated with fibrosis in seven different loci [19]. Three CpG sites were located in genes, and four were located in enhancers. We extracted the genes and genes linked to the enhancer for pathway analysis. In the same study, the authors also used a reference-based method of estimating cell-type proportion based on DNAm data EpiDISH and found that the proportion of natural killer cells increased, while epithelial cell proportions decreased with disease stage.

#### 3.2.2. Differentially Methylated Genes in ALD

One study performed motif enrichment analysis of different methylated regions to identify transcriptional regulators in alcoholic hepatitis (AH) [20]. To perform this analysis, they first identified a thousand differentially methylated CpGs between AH and normal livers in an epigenome-wide analysis. They found a parallel relationship between hypermethylation and down-regulation of regions controlled by liver-enriched transcription factors such as HNF4α, HNF1α, CEBPα, SREBPs, CEBPβ and anti-fibrogenic factors such as peroxisome proliferator-activated receptor (PPARγ) livers (we extracted those for our further pathway analysis).

#### 3.2.3. Differentially Methylated Genes in MASLD and ALD

Only two studies simultaneously examined MASLD and ALD [27,28]. One study performed a target analysis of anti-fibrogenic genes (***PPARα*** and ***PPARγ***) and three genes known to drive fibrogenesis (***TGFβ1***, ***Collagen 1A1*** and ***PDGFα***) [27]. They performed two separate analyses: MAFLD with mild fibrosis vs. MAFLD with severe fibrosis and ALD vs. a “normal” liver. Anti-fibrogenic genes were hypomethylated, and ***TGFβ1*** and ***PDGFα*** were hypermethylated in mild fibrosis in MAFLD compared to severe fibrosis, and the same was found for normal livers compared to ALD. Notably, there was no direct comparison between ALD and MASLD, but the methylation status of a panel of fibrosis-related genes was similar in both liver injury diseases. In another study, a target analysis of l-DNAm of genes related to the Ufmylation pathways (which are related to the formation of Mallory–Denk bodies) found that ***Ufm1***, ***Ufc1*** and ***UfSP1*** were hypermethylated in ASH and NASH when compared to normal subjects [28].

### 3.3. MASLD Pathways, ALD Pathways, Common Pathways MASLD and ALD

#### 3.3.1. MASLD Pathways

We identified nine pathways related to hypermethylated genes and forty-four pathways related to hypomethylated genes. The pathways involving the highest number of differentially methylated genes were the pathways in cancer, multiple pathways in the group of “metabolic pathways”, pathways of focal adhesion, and the PI3K-signaling pathway. See Supplementary Table S2 for all pathways.

#### 3.3.2. ALD Pathways

Twenty pathways related to hypomethylated genes and fifty-one to hypermethylated genes were identified. Pathways in cancer, autophagy, apoptosis, cellular senescence, and MTOR signaling are among them. See Supplementary Table S2 for all pathways.

#### 3.3.3. Common Pathways in MASLD and ALD

Sixteen pathways were related to differentially methylated genes in both diseases: pathways in cancer, “metabolic pathways”, PI3K-Akt, the AMPK signaling pathway and microRNAs in cancer are among them. See Table 2 for the common pathways.

### 3.4. Risk of Bias Assessment

The level of heterogeneity was high and the bias analysis showed that the risk of bias was moderate to severe. Five articles were deemed to be at serious risk of bias, while the remaining ten had a moderate risk. Most articles did not employ methods to balance all the key characteristics between groups, leading to differences among them. Regarding BMI, some studies did not include normal-weight subjects. Furthermore, participants were initially recruited for studies with other primary objectives, and only later were their data analyzed for DNA methylation. Additionally, there is a challenge in measuring the type of alcohol consumed by patients.

**Figure 2:**
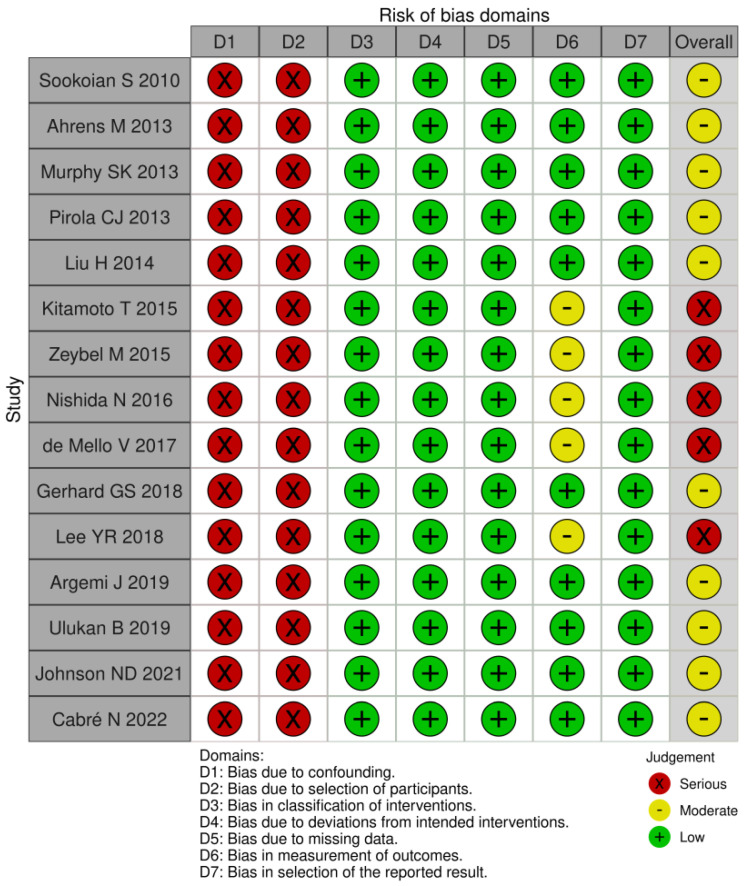
ROBINS-I risk of bias in non-randomized studies of interventions [18,19,20,21,22,23,24,25,26,27,28,29,30,31,32].

Confounding bias (D1) arises when a pre-intervention prognostic factor influencing the likelihood of exposure is present. Selection bias (D2), on the other hand, occurs due to systematic imbalances between groups that result from the recruitment process. Therefore, based on the above findings, we determined that all articles had a serious risk of bias due to confounding and the selection of participants. Regarding all other domains (D3–D7), we judge that most articles had a low risk of bias; this assessment stems from the fact that these domains pertain to the methodology employed in the studies after patient selection. See Figure 2. However, we want to highlight that the ROBINS-I tool was not designed for this specific question, so our bias evaluation should be interpreted with caution.

## 4. Discussion

This systematic review is the first that summarizes DNA methylation patterns and highlights the complexity of the epigenetic landscape in ALD and MASLD, underscoring how these patterns reflect not only the commonalities but also the unique pathways that distinguish these two conditions. While both diseases involve metabolic dysregulation and inflammation, their distinct DNA methylation profiles suggest that they engage different cellular processes and molecular networks.

**Figure 3:**
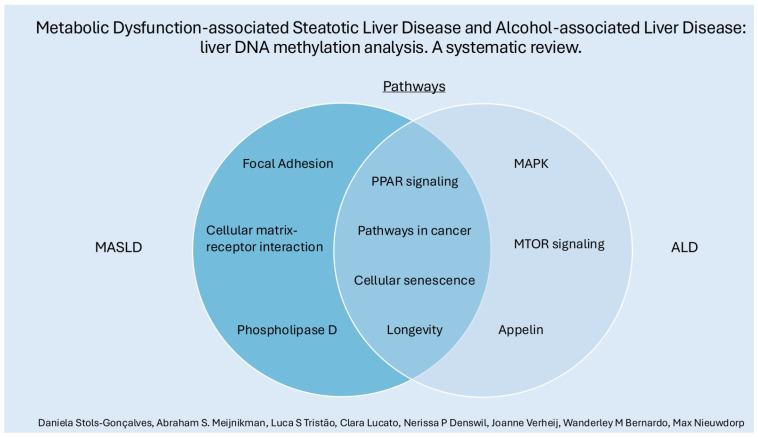
Some (individually and shared) pathways related to differentially methylated genes in MASLD and ALD.

Differential methylation patterns in some pathways were observed in both MASLD and ALD, such as PPAR signaling, cellular senescence, cancer-related pathways, and longevity. See Figure 3. Despite sharing some overarching characteristics, these diseases may take distinct routes in their pathophysiology. MASLD displays more significant associations with pathways like focal adhesion, extracellular matrix–receptor interaction, and phospholipase D. In contrast, ALD is more prominently linked to differentially methylated genes in pathways such as MAPK and apelin signaling, underscoring the possibility of unique molecular mechanisms driving each disease.

The peroxisome proliferator-activated receptor genes PPARα and PPARG are differentially methylated in both diseases [27]. Interestingly, the PPAR signaling pathway was the second most significant pathway related to the seven hypermethylated genes in ALD. PPARs are transcription factors activated by ligands belonging to the nuclear receptor family. Their function in MASLD has been exhaustively reviewed, and all three PPAR isotypes (PPARα, β/δ, and γ) regulate lipid metabolism [33]. In particular, PPARα plays a role in both MASLD and ALD; its expression is negatively correlated with NASH severity, and treatment with a PPARα agonist reverses abnormalities in ethanol-fed mice [34,35].

“Pathways in cancer” appear as the most significant common pathways related to hypomethylated genes in ALD and MASLD, while metabolic pathways appear as the most significant hypermethylation-related pathways in both diseases. Moreover, one of the most serious complications of both ALD and MASLD is the risk of progression to HCC. One of our findings was that the PI3K-Akt signaling pathway was differentially methylated in both diseases, a pathway strongly related to the pathogenesis of most cancers, including tumorigenesis and HCC progression [36,37].

Cellular senescence is a state of cell-cycle arrest characterized by the upregulation of cyclin-dependent kinase inhibitors, such as p16 and p21, and is recognized as one of the hallmarks of aging [38]. Cellular senescence has important physiological functions, and, although necessary to avoid oncogenic processes, it can also contribute to the development of diseases such as MASLD, as previously described [39]. The senescence pathway was one of the findings in the hypermethylated genes related to the ALD pathways. Senescent cells release cytokines, growth factors, and matrix-remodeling enzymes, collectively known as the senescence-associated secretory phenotype (SASP), which represents a second fundamental aspect of cellular senescence and can lead to tissue dysfunction through paracrine signaling. This state can be triggered by various cellular stressors, including telomere dysfunction and oxidative stress. In the context of liver disease, cellular senescence has been identified in hepatocytes, cholangiocytes, stellate cells, and immune cells across multiple chronic liver conditions. Increased cellular senescence has also been noted in the livers of patients with ALD, correlating with disease severity.

Interestingly, “longevity-regulating pathways” were also among the common pathways related to hypermethylated genes in both diseases. DNA methylation is a possible mechanism involved in fine-tuning the optimal senescence mechanism, requiring equilibrium to avoid opposing situations such as excessive cell death or excessive cell proliferation.

In MASLD, hypermethylated genes were annotated to the “insulin signaling pathway”. The insulin pathway was not found to be amongst the most important pathways in ALD. Although the correlation between MASLD and IR is widely known, whether IR plays a role in ALD has not yet been fully elucidated. Ceramide metabolism, toll-like receptor 4 (the lipopolysaccharide cell surface receptor for lipopolysaccharide), and inhibition of insulin-responsive genes are among the possible mechanisms involved in IR and ALD [40,41,42]. In contrast, a systematic review and meta-analysis on the effect of moderate alcohol consumption on insulin sensitivity concluded that alcohol consumption can improve insulin sensitivity in women [43]. However, recent studies have shown that moderate alcohol intake is associated with an increased risk of death in more than 10,000 people with cardiometabolic risk factors [44]. At the gene level, ***IGF1*** is hypermethylated in ALD. IGF-1 has been proposed as an indirect marker of hepatic insulin resistance. Whether l-DNAm of ***IGF1*** plays a role in IR in ALD remains to be elucidated [45].

The second most important MASLD pathway related to hypomethylated genes was focal adhesion, and in the top 12, we also observed the extracellular matrix (ECM)–receptor interaction pathway. In MASLD-related fibrosis, liver sinusoidal endothelial cell (LSCE) dysfunction leads to the production of profibrogenic molecules (laminin, TGFβ, Hedgehog molecules, and fibronectin), which activates hepatic stellate cells, causing excessive deposition of the extracellular matrix [46].

Interestingly, the phospholipase D signaling pathway was one of the most significant MASLD pathways related to hypomethylated genes. Phospholipase D, an enzyme that catalyzes the hydrolysis of phosphatidylcholine into phosphatidic acid and free choline, has been shown to be a potential modulator of metabolic syndrome, diabetes and obesity [47,48]. Phospholipase D1-deficient mice develop MASLD via an autophagy defect [49]. To our knowledge, there are no studies in humans that are specific to the role of phospholipase D in MASLD.

Some of the most important pathways in ALD are related simultaneously to both hypermethylated and hypomethylated genes, such as the MAPK signaling pathway, autophagy, endocytosis, and the apelin signaling pathway. Ethanol-induced activation of MAPK signaling has been previously studied, and, here, we see that DNA methylation maybe involved in this process [50]. The apelin signaling pathway has been previously associated with hepatic stellate cell activation and liver fibrosis [51].

Our study has several limitations, as all included studies had a moderate to serious risk of bias; thus, our findings should be interpreted with caution. In addition to the well-known main culprits of confounding and bias in DNA methylation studies, such as age, sex, BMI, tobacco use, concomitant diseases, and co-medication, we can also add alcohol consumption, especially binge drinking, which can cause acute changes in inflammatory markers, making it very difficult to have a standard profile in ALD [52]. We also do not know how much of the differential DNAm is related to the change in cell proportions, and not to the disease itself. DNA methylation arrays differed between studies. Single-cell DNA methylation may offer a better understanding of different steatotic, inflammatory, and fibrogenic elements in the future. Characterization of DNA methylation in the early stages of ALD remains a challenge, as most patients already have severe fibrosis or cirrhosis at the first histological diagnosis.

It is important to note that our review focuses specifically on DNA methylation, excluding other epigenetic factors such as histone modifications and non-coding RNAs, which have been previously studied under both conditions [53].

Pathways in cancer were among the most important common differentially methylated pathways in ALD and MASLD. Therefore, more research is needed to determine the potential role of l-DNAm as a marker for the risk of HCC. Additionally, l-DNAm in the insulin signaling pathway in MASLD and the MAPK signaling pathway appeared among the most significantly differentially methylated pathways. The role of DNA methylation in senescence and longevity also requires further investigation.

In conclusion, the role of l-DNA methylation in MASLD and ALD is multifaceted, influencing numerous biological pathways that underpin disease progression, fibrogenesis, and carcinogenesis. The translational potential of these findings lies not only in advancing our understanding of liver disease mechanisms but also in developing predictive biomarkers and targeted therapies for better patient management, since this is still lacking. As the field of epigenetics continues to evolve, future research should aim to clarify the interplay between DNA methylation and other epigenetic mechanisms, with a focus on the development of specific epigenetic therapies that address the unique molecular signatures of MASLD and ALD. Through continued exploration of these epigenetic landscapes, we move closer to precision medicine approaches that could transform the management and outcomes of chronic liver diseases.

## Figures and Tables

**Table 1:** Included studies: DOI, first author, year of publication, patient population, sample size, age, sex and BMI from the 15 included papers. Abbreviations: NAFLD (nonalcoholic fatty liver disease), ALD (alcohol associated liver disease), NASH (nonalcoholic steatohepatitis). “A” and “B” are different groups that were compared in each study.

Study Number	Author	Year	Patient Population		Sample Size		Age (Year)		Sex (Fem/Male)		BMI	
			A	B	A	B	A	B	A	B	A	B
1	Cabré N	2022	Obese NASH (NAS ≥ 5)	Obese non-NASH (NAS ≤ 2)	8	8	Matched but not specified for this subgroup		Matched but not specified for this subgroup		Matched but not specified for this subgroup	
2	Johnson ND	2021	G3 = grade 3 fib G3–4 = grade 3–4 fib G4 = grade 4 fib	G0 = no fibrosis	G3 n = 55 G3–4 n = 36 G4 n = 28	206	G3 49.5 ± 10.0 G3/4 50.4 ± 8.1 G4 53.8 ± 9.5	(All 48.6 ± 11.5) G0 47.2 ± 12.4	G3 61.8% G3/4 80.5% G4 78.5%	(All 77%) G0 80.5%	G3 48.6 ± 8.0 G3/4 46.5 ± 9.7 G4 48.1 ± 12.1	All 47.1 ± 9.1 G0 46.6 ± 8.8
3	Argemi J	2019	AH	Normal liver	6	5	-		-		-	
4	Ulukan B	2019	NASH	NAFLD F0	Cirrhotic NASH (f4) n = 22 (F1/F2 F3) n = 24	9	-		-		-	
5	Gerhard GS	2018	NAFLD cirrhosis	Normal liver	11	15	47.1 ± 6.3	51.3 ± 8.2	100% women		41.0 ± 4.6	44.4 ± 6.6
6	Lee YR	2018	NAFLD	Normal liver	54	18	-		-		-	
7	de Mello V	2017	Obese NASH	Obese Normal liver Obese SS	26	Normal n = 35 SS n = 34	NASH = 51.3 ± 7.9	Nl = 50.7 ± 7.0 SS = 46.9 ± 7.6	13/13	Nl (11/24) SS (10/24)	NASH 43.4 ± 6.4	Nl 42.4 ± 6.1 SS 43.5 ± 4.7
8	Nishida N	2016	NAFLD	HCC or normal liver	65	16	54.3 (50.7–57.9)		40/25	-	-	29.3 (28.0–30.6)
9	Kitamoto T	2015	advanced fibrosis (F2–4)	Mild fibrosis (F0–1)	29	36	-		-		-	
10a	Zeybel M	2015	advanced fibrosis (F2–4)	NAFLD, minimal fibrosis	9	8	51.88 (40.43–63.32)	60.56 (54.5–66.61)	100% male		35 (30–43.2)	36 (30–46)
10b	Zeybel M	2015	ALD	Normal liver	10	17	63.31 (67.94–54.71)	46.3 (39.09–57.27)	10 male (58%)	7 male (70%)	24.2 (19–32.5)	
11	Hui Liu	2014	NASH	Alcoholic hepatitis Normal liver	AH 3–5 NASH 3–5	3	-		-		-	
12	Pirola CJ	2013	NAFLD (simple steatosis and NASH)	Near normal histology	22	Non-MS almost nl n = 18 Steatosis n = 23	NASH 48 ± 9.2	Nl 48.3 ± 8.7 Stea 51.6 ± 10.5	NASH 12/10	Fem/male Nl 10/8 Steat 14/9	NASH 31.2 ± 6	Nl 25.3 ± 4.1 Steat 31.6 ± 5
13	Ahrens	2013	NASH	No adv Normal control “Healthy” obese Steatosis	15	Obese healthy n = 18 Steat n = 12 Normal control n = 18	44 (41–50)	46 (37–49) 47 (40–50) 51 (44–72)	Male % 0	Male % 42 27 50	45 (42–49)	50 (47–55) 49 (44–56) 24 (21–26)
14	Murphy SK	2013	Advanced NAFLD F3–F4	Mild NAFDL F0–F1	23	33	Advanced 51.7 ± 10.3	Mild = 51.5 ± 10.3	Advanced 5/18	Mild 12/21	Advanced 33.8 (31.3–41.9)	Mild 32.8 (28.4– 40.4)
15	Sookoian S	2010	NAFLD	NAFLD Control (no histological fatty changes)	63	11	NAFLD 50.3 ± 9.9	49.46 ± 10.2	NAFLD 32/31	Control 5/6	NAFLD 31.9 ± 5.6	Control 27.9 ± 5.9

For the complete list of all extracted data, see also Supplementary Table S1.

**Table 2:** KEGG common pathways between ALD (alcohol-associated liver disease) and MASLD (metabolic dysfunction-associated liver disease) with at least 3 differentially methylated genes in alphabetical order.

Pathways	Number of Genes with Changes in Methylation			
	Hypo ALD	Hyper ALD	Hypo MASLD	Hyper MASLD
AMPK signaling pathway		4		3
Amoebiasis		3	6	
Growth hormone synthesis, secretion and action		4	3	
Human papillomavirus infection	5		6	
Lipid and atherosclerosis	3	4	3	
Longevity-regulating pathway		4		3
Metabolic pathways	3	34	6	9
MicroRNAs in cancer		3	3	4
Neutrophil extracellular trap formation		3	3	
Pathogenic Escherichia coli infection	3		3	
Pathways in cancer *	7	6	11	7
PI3K Akt signaling pathway	3	3	7	
Regulation of actin cytoskeleton	5		3	
Thermogenesis		5		3
Tight junction		3	4	
Transcriptional dysregulation in cancer		3		3

* In pathways in cancer, the ***PPAR*** was differentially methylated in ALD and MASLD.

## Supplementary Materials

The following supporting information can be downloaded at https://www.mdpi.com/article/10.3390/cells13221893/s1: Table S1: All data extraction, Table S2: All pathways mythylation.
